# Supplementary material for: Sodium-Calcium Exchanger 2: A Pivotal Role in Oxaliplatin Induced Peripheral Neurotoxicity and Axonal Damage?
Source: Int J Mol Sci. 2022 Sep 2;23(17):10063. doi: 10.3390/ijms231710063 (PMC9456447; doi:10.3390/ijms231710063)
Supplement: Supplementary file 1 [file ijms-23-10063-s001.zip › ijms-1885185-supplementary.pdf]

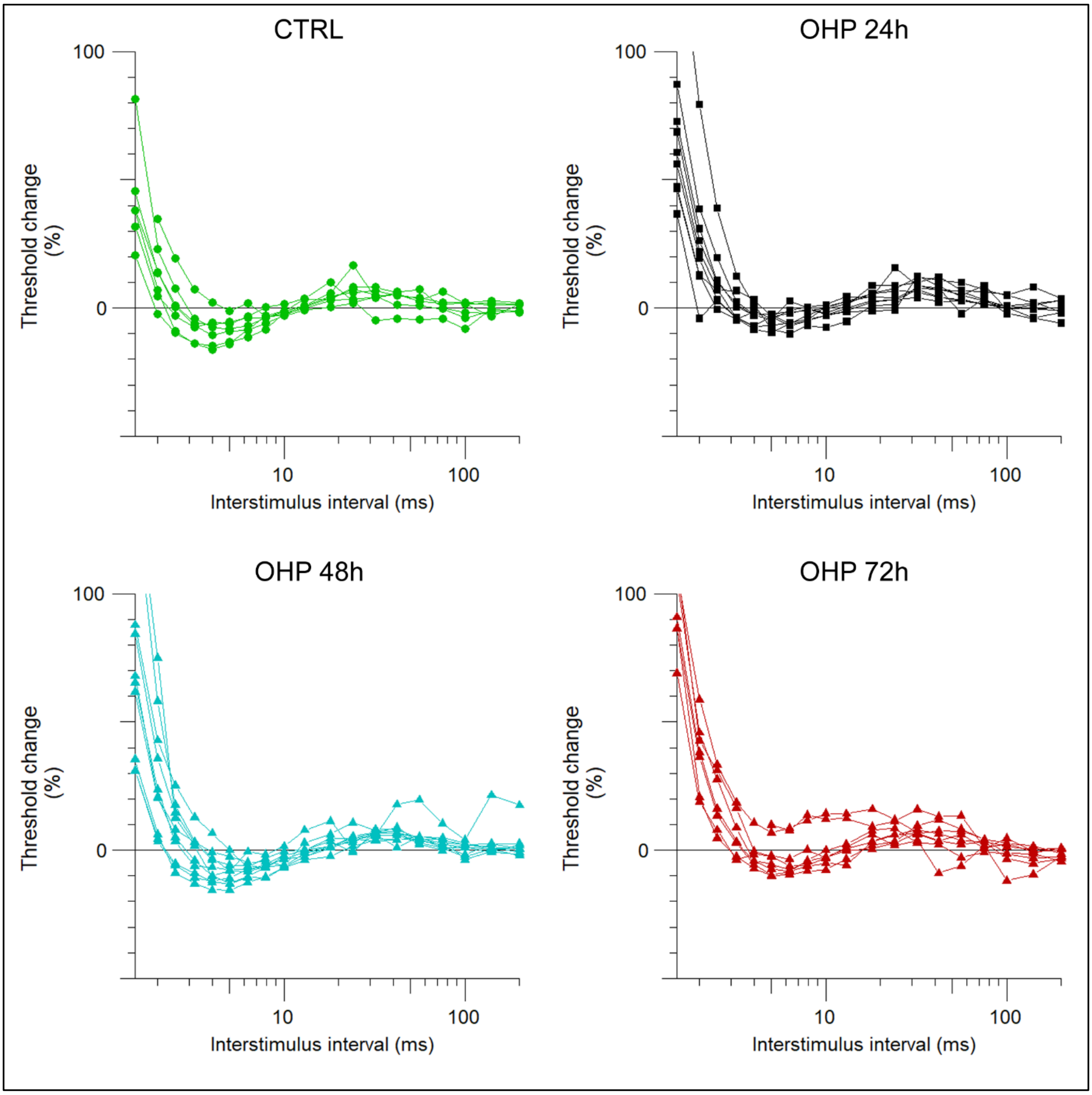

Supplementary Figure S1. Exemplificative traces of single recordings for each group are shown (recovery cycle of nerve excitability testing). CTRL: control group; OHP: oxaliplatin.
